# Implementation determinants of risk-stratified gestational diabetes mellitus screening in community-based women’s peer groups in rural western Kenya

**DOI:** 10.1186/s12884-026-09081-6

**Published:** 2026-05-18

**Authors:** Rachel Ogumbo, Violet Naanyu, Laura Ruhl, James Akiruga, Evelyn Kaluhi, Sheilah Chelagat, Hector Nyaboga, Sonak Pastakia

**Affiliations:** 1 Academic Model Providing Access to Health care, Eldoret, Kenya; 2 School of Arts and Social Sciences, Department of Psychology, Anthropology, and Sociology, Moi University, Eldoret, Kenya; 3 College of Pharmacy, Purdue University, West Lafayette, IN USA; 4 School of Medicine, Department of Medicine, Indiana University, Indianapolis, IN USA; 5 College of Health Sciences, Moi University, Eldoret, Kenya; 6 College of Medicine, Department of Pharmacology, Moi University, Eldoret, Kenya

**Keywords:** Gestational diabetes mellitus, Risk-based screening, Barriers and facilitators, Theoretical domains framework, Community-based peer groups, Rural western Kenya

## Abstract

**Background:**

Gestational Diabetes Mellitus (GDM) contributes to maternal and neonatal complications and is a growing concern in Kenya. The 2-hour oral glucose tolerance test (OGTT) is recommended to diagnose GDM between 24 and 28 weeks of pregnancy, but uptake remains low in settings experiencing financial and structural barriers to health care access. The Stratification Risk of Diabetes in Early pregnancy (STRiDE) tool is a risk-prediction model designed to identify women at elevated risk for GDM. The STRiDE-GDM screening approach integrates this tool within community-based peer groups to guide targeted, risk-stratified GDM screening and referrals to local health care facilities. However, little is known about implementation of STRiDE-GDM in Kenya. This study explored determinants (facilitators and barriers) of STRiDE-GDM implementation among pregnant and postpartum women in western Kenya.

**Methods:**

Community Health Promoters (CHPs) recruited 18 women from *Chamas*, a community-based women’s peer group program to complete in-depth interviews exploring beliefs, perceptions, and experiences related to the STRiDE-GDM screening approach. Transcribed audio-recordings were translated from Swahili to English. We conducted thematic analysis using a hybrid inductive-deductive coding approach to identify and explore facilitators and barriers to STRiDE-GDM implementation. The resulting themes were then systematically mapped to the Theoretical Domains Framework (TDF) domains.

**Results:**

Participants had a mean age of 26.6 years (SD = 7.2); 39% were pregnant, with most experiencing their first pregnancy and one-half were enrolled in the national health insurance. Key implementation determinants were mapped to relevant domains within the TDF. Facilitators to engage in the STRiDE-GDM screening included motivations to maintain maternal health in order to fulfill caregiving responsibilities, perceived benefits of early GDM diagnosis, support from *Chamas* peer groups and guidance from trusted CHPs. Barriers included limited awareness about GDM screening, transportation and cost constraints, competing household responsibilities, stigma, disclosure concerns within the *Chamas* peer groups, limited spousal support, and fear of adverse outcomes.

**Conclusion:**

Findings highlight that individual, social and structural factors influence engagement in STRiDE-GDM screening. Community-based peer groups, such as *Chamas* emerged as an ideal setting to support screening. Addressing modifiable barriers including low GDM screening awareness, transportation, cost, and scheduling constraints prior to implementation may improve feasibility and uptake.

**Supplementary Information:**

The online version contains supplementary material available at 10.1186/s12884-026-09081-6.

## Introduction

Gestational Diabetes Mellitus (GDM) is a condition defined as maternal hyperglycemia first noted during pregnancy [1]. GDM is a growing concern affecting an estimated 14% of pregnancies worldwide. This number varies between 7% and 28% depending on the population studied and the GDM diagnostic criteria used [2, 3]. Maternal complications experienced from GDM include pre-eclampsia, postpartum hemorrhage, intrauterine infection, and caesarian delivery [4–6]. In neonates, GDM also increases the risk for stillbirth, preterm delivery, respiratory distress syndrome and congenital malformation [4–6]. In the long-term, offspring born to mothers with GDM have a higher likelihood of developing diabetes in the future [4–6]. Additionally, up to 50% of women who develop GDM in pregnancy, progress to develop Type 2 diabetes within 5 to 10 years [7].

Approximately 90% of reported GDM cases occur in Low- and Middle-Income Countries (LMICs), including sub-Saharan Africa. In these settings, limited resources and low screening coverage hinder timely diagnosis and management [8]. In Kenya, GDM prevalence data are limited and highly variable ranging from approximately 3% in community-based studies to over 11% in urban referral settings [9, 10]. This variability depends on the type of screening, diagnostic criteria and disparities between urban and rural settings [6, 9]. Nationally, about 4% of pregnancies are affected by hyperglycemia, highlighting the relevance of GDM within maternal health and the growing Type 2 diabetes burden [11].

This growing burden is compounded by limited access to primary health care services, which are critical for both maternal and chronic disease management. Although these services exist in rural western Kenya, as in other LMICs, access remains limited [12]. As a result, the first-time women engage with a formal health system is often during pregnancy, creating a critical window for intervention. Even when women access primary care or antenatal care (ANC), conditions like GDM may go undetected without structured screening and adequate resources. Community-based peer group programs such as “*Chamas for Change (Chamas)*” can help bridge this access gap by providing education, support, and access to screening during this critical life stage.

*Chamas* is a community-based program in rural western Kenya, led by community health promoters (CHPs) who support pregnant and postpartum women [13–16]. *Chamas*, a Swahili word meaning “group”, clusters pregnant women together at the start of their pregnancies and provides a platform for health education, peer support, and opportunities for microfinance engagement [13–16]. Chamas peer groups can therefore serve as a community-based service delivery platform to screen for chronic diseases such as GDM and ensure improved linkage into the primary health care systems.

Despite recommendations on early screening and diagnosis for effective GDM management, this practice is missed during most ANC visits [17–19]. Challenges to adequate GDM screening in LMICs include limited access to primary care, lack of uniform screening strategies, complexity of performing guideline recommended oral glucose tolerance tests (OGTT) and cost [9, 17, 19, 20]. These challenges limit screening in public facilities putting women and infants at risk of preventable consequences from undiagnosed GDM [9, 17, 19, 21]. Providing universal OGTT for all pregnant women presenting for care would further burden an already overextended system.

To address these gaps, the Stratification of Risk of Diabetes in Early Pregnancy (STRiDE) risk prediction model was developed to estimate GDM risk in early pregnancy (< 20 weeks’ gestation) and, only target high-risk women for the resource intensive OGTT between 24 and 28 weeks’ gestation [20]. The STRiDE tool uses a point-of-care HbA1c as its core predictor in combination with other maternal factors to identify women at high risk for GDM in early pregnancy. Because the device is portable, it can be implemented in rural community settings by trained CHPs. GDM risk-stratification is therefore initiated in the community. Medium-and- high-risk participants who receive a positive GDM diagnosis following the OGTT are subsequently referred to the local health facility for care typically provided by clinical officers. Implementing effective GDM screening using the STRiDE tool thus requires collaboration among key stakeholders including pregnant and postpartum women, CHPs and clinical officers (Supplementary Materials S1).

Despite the STRiDE’s tool potential to improve GDM screening in low-resource settings, little is known about factors that may influence its implementation. Guided by the Theoretical Domains Framework (TDF), this study aimed to explore facilitators and barriers to implementing the STRiDE tool in *Chamas* peer groups through stakeholder interviews. The modifiable barriers will be disseminated back to local stakeholders, who will be invited to collaborate and share recommendations in developing solutions to address them. These recommendations will guide revisions to the STRiDE-GDM implementation strategy. The revised strategy will be piloted in *Chamas* peer groups to assess feasibility and inform strategies to support GDM screening uptake.

## Methods

### Study setting and participants

This study took place in Bungoma County in western Kenya. Bungoma is a rural setting which has a population of approximately 2 million people [22]. Bungoma residents experience poor maternal and newborn health outcomes compared to the national average [22]. We leveraged “*Chamas for Change (Chamas)*” community-based peer groups that support pregnant and postpartum women which are common in this setting. Chamas groups support pregnant women together at the start of their pregnancies and provide a platform for health education, peer support, and opportunities for microfinance engagement [13–16].

The key stakeholders for this study included Chamas participants, CHPs and Clinical officers who were recruited to capture diverse perspectives on determinants of the STRiDE-GDM screening approach as highlighted in (Supplementary Materials 1). The present manuscript presents findings from Chamas participants, who were the core participant group. Insights from CHPs and Clinical officers will be presented in subsequent publications.

Eligible Chamas participants were between 18 years of age to 50 years of age. Participants were excluded if they had a pre-existing diagnosis of type 1 or type 2 diabetes, severe anemia (Hemoglobin < 8 g/dl), sickle cell anemia, were taking any medications that affected glycemic control or were unable to consent or complete study protocol. A total of 18 women participated in the in-depth interviews, including women pregnant for the first time, women who had been pregnant before, and postpartum women who had been screened for GDM previously as part of routine care (not as part of a structured research study). Stratification was employed to capture a wide range of women’s beliefs of the proposed STRiDE-GDM screening approach.

### Study design

This study represents the first, qualitative phase of an exploratory sequential mixed-methods project. In this phase, participants explored facilitators and barriers of engaging in the proposed STRiDE-GDM screening. Qualitative findings were then used to inform modifications of the implementation plan prior to feasibility piloting. Our methods were guided by the TDF-implementation research process described by Atkins et al. [23]. In this process the authors use the TDF to systematically identify, prioritize and explain behavioral factors that could likely influence implementation processes. Key TDF domains are then mapped to behavior change techniques or highlighted as priority areas for consideration during intervention refinement prior to a feasibility pilot study. The TDF is a widely used framework that synthesizes 33 behavioral theories and summarizes them into 14 behavior change domains [23]. This framework has previously been applied in GDM studies to identify determinants that influence health-related behaviors such as uptake of postpartum diabetes screening after GDM diagnosis and adherence to GDM management recommendations post diagnosis [24, 25].

### Recruitment and sampling

Eligible participants were identified through purposive sampling with assistance from an administrator who organizes the *Chamas* peer groups and CHPs. Eighteen participants who expressed interest and met inclusion criteria were subsequently recruited by CHPs in person or via telephone. This is consistent with prior qualitative research studies which suggest that thematic saturation if often achieved around 10 interviews while meaning saturation occurs around 16 to 24 interviews [26]. Data were collected from all 18 participants, and the emergence of new insights was monitored throughout the analysis. No new beliefs or themes emerged in the final interviews, confirming that thematic saturation was achieved.

### Data collection section

Semi-structured interviews (SSIs) were conducted with all 18 participants between December 2024 and January 2025. SSIs explored beliefs, experiences and perceptions related to the target behavior for this study which was engagement in risk-stratified GDM screening using the STRiDE tool (STRiDE-GDM screening). Throughout the manuscript, “STRiDE” refers to the risk-prediction tool, while “STRiDE-GDM” refers to the broader screening approach that integrates the tool within *Chamas*, a community-based women’s peer group platform. The STRiDE tool is a composite risk prediction model that incorporates point-of-care HbA1c as its core predictor, in addition to other maternal risk factors to estimate a woman’s likelihood of screening positive for GDM [20]. Risk-stratification is initiated in early pregnancy (< 20 weeks’ gestation) by trained CHPs in *Chamas* peer groups. Medium- and high-risk participants receive confirmatory OGTT diagnostic testing between 24 and 28 weeks’ gestation. Those who test positive for GDM are referred to the nearest health facility through an established referral pathway to obtain treatment. Participants exposure to risk-based GDM screening varied. Postpartum participants had previously experienced a similar screening as part of routine care, while all others had not. All participants shared their beliefs and perceptions of the proposed STRiDE-GDM screening process rather than undergoing the intervention which was planned for a future pilot feasibility study.

Interviews were guided by a 13-question SSI topic guide informed by the TDF. To confirm that questions were understandable and relevant, the SSI guide was reviewed by the research team and a qualitative expert, then piloted for face validity with 3 *Chamas* participants. Face validity, referring to whether questions were clear and that the translated Swahili guide accurately captured the intended meaning of each item was assessed. Revisions were made to simplify complex questions, improve clarity and shorten the length of the interview. Data from the SSI pilots were not included in the final analyses. The final version of the SSI guides for the *Chamas* participants is included. (Supplemental Materials S2)

Interviews were scheduled at the convenience of *Chamas* participants and conducted in their home setting, at nearby health dispensaries, or at hospitals close to their homes. All three interviewers (two female, one male) were deeply knowledgeable about maternity and child health services in this community after having worked with the *Chamas* program for many years. As a result, some interviewers previously engaged with participants, but none provided clinical care. Prior to each interview, participants received a standardized overview of the proposed STRiDE-GDM screening approach, delivered verbally during a group session and reinforced during the informed consent process. This overview explained the study purpose, the STRiDE-GDM screening process, and clarified that the interviews would focus on participants’ experiences, beliefs and anticipated perceptions. During consent, interviewers also provided a written description of the proposed STRiDE-GDM screening approach to ensure shared understanding. Participants were encouraged to ask questions, and clarification was provided as needed.

Written informed consent was obtained from all participants, with the option to decline questions or withdraw at any time. Following consent, interviewers collected basic demographic data for each participant prior to the start of the interviews which were all conducted in Swahili and audio recorded with permission. Interviews lasted approximately 20 to 25 min on average. The interviews consisted of broad open-ended questions such as “Have you ever been screened for GDM? If so, what challenges did you face in being screened? What aspects about the screening process were helpful?” The open-ended questions elicited perceived barriers that could be modified and facilitators that could be leveraged for successful implementation of the STRiDE-GDM screening approach. Domain-informed prompts elicited broader explanations of participants’ experiences and perceptions to STRiDE-GDM screening, as well as potential targets to improve uptake of the intervention [23, 27].

Participants were renumerated with 500 Kenya shillings (USD 4) for transport reimbursement. To enhance trustworthiness and address interviewer positionality, interviewers were encouraged to take notes during and after each interview, reflecting on and documenting their perceptions or reactions. Team debriefings were also held at the end of each interview day to discuss observations and interpretations. These practices helped to mitigate the potential for bias on data collection processes.

Completed interviews were downloaded digitally into a password-protected system and assigned a unique code. Original participant identifiers reflected prior exposure to a similar screening process in routine care (T= prior exposure; W = no exposure). Since coding and analysis were collective, these identifiers were not used, and participant quotes are presented using standardized IDs [P1-P18]. Audio recordings were then transcribed verbatim in Swahili and translated into English. Translation enabled all team members access and code responses deductively using the TDF, which is designed and applied in English. To assess for accuracy and completeness of transcribed and translated data, the interviewers conducted time checks on transcripts by verifying that different time points in the audio recording aligned with the corresponding transcribed interview text.

### Data analysis section

Transcripts were imported into Microsoft Excel for manual coding. Data were coded by the analytic team, consisting of two trained research assistants and post-doctoral fellow with expertise in qualitative methods. The analytic team applied a hybrid inductive-deductive coding approach informed by the TDF to guide identification of facilitators and barriers to engagement in the STRiDE-GDM screening [28].

At first the analytic team independently read the transcripts and generated preliminary inductive codes before engaging with the TDF codebook. The blind reading enabled the analytic team identify other codes that may not have been in the structured TDF codebook to enhance the inductive approach [23]. The draft codebook was then refined and piloted through double-coding of one transcript [23, 27]. Piloting a codebook has been highlighted as important to reduce subjective bias when multiple coders work together [23, 27]. New codes were incorporated into the codebook as they emerged for deductive analysis. After reaching consensus on the pilot transcript, three team members independently coded two additional transcripts and discussed discrepancies to refine the coding approach [25, 27]. This process strengthened credibility of the analysis and informed coding of the rest of the interviews. Data were then analyzed using the TDF in a systematic, iterative process in which coding of early transcripts informed refinement of codes and identification of themes in subsequent transcripts.

Belief statements were coded to one or more TDF domains when they reflected multiple influences on engagement in STRiDE-GDM screening. Double-coding was allowed to capture this complexity with domain assignment guided by the TDF definitions. Discrepancies were discussed and resolved within the analytic team. For example, participants’ experiences of long queues while fasting were coded to Environmental Context and Resources (structural barrier), Reinforcement (negative experience), and Emotions (affective response), reflecting the multiple ways the experience could influence engagement in the STRiDE-GDM screening. New insights were monitored throughout the analysis. Codes within each domain were then grouped into broader themes, which were categorized as facilitators or barriers. No new beliefs or themes emerged in the final interviews, indicating that thematic saturation was achieved. The team reviewed theme frequency, refined themes to align with relevant TDF domains, and selected illustrative quotes for each.

The salience of each domain for engagement in the proposed STRiDE-GDM screening was assessed using three criteria adapted from prior TDF studies: (i) frequency, the relative number of distinct belief statements related to the STRiDE-GDM screening approach within each domain was counted across participants and presented descriptively as numeric values; (ii) conflicting beliefs, domains were coded as present or absent (Yes/No) based on whether participants expressed opposing beliefs that both enabled and hindered engagement in the STRiDE-GDM screening; and (iii) Perceived influence on GDM screening behavior was determined by the research team based on participant narratives and TDF theory and rated as low, moderate, or high [23, 24, 27]. Overall importance was then determined by combining assessment from these three criteria. The analytic team discussed and reached consensus on these assessments to ensure credibility (Table 2). Of note, Frequencies reflect the number of distinct belief statements reported across participants within each TDF domain. If a participant expressed the same belief in response to different questions, each instance was counted separately. For example, if a participant reported that travel to the facility was difficult for both initial GDM screening and referral to care, each response was counted separately to capture context-specific relevance. Repeated mentions of the same belief within a single response were only counted once. In addition to frequency, conflict, and perceived influence on GDM screening, we also considered the feasibility of addressing specific beliefs within each domain prior to implementation of the pilot study.

Although participants were purposively recruited across pregnancy and postpartum phases to cover diverse perspectives, data were analyzed collectively rather than by each separate stage. To enhance trustworthiness, multiple strategies were employed, including use of trained research assistants familiar with study context, audio recording and transcript accuracy checks, team-based coding with regular consensus discussions. Interviewer positionality and prior experiences with *Chamas* program were discussed during analysis to minimize interpretive bias. We adhered to recognized criteria and standards for reporting qualitative research, including the COREQ checklist [29, 30] (Supplemental Materials S3). An audit trail of the coding process was also maintained to ensure the credibility and trustworthiness of the study [29, 30].

## Results

### Participant characteristics

18 *Chamas* women were interviewed. *Chamas* participants’ mean age was 26.6 years (SD = 7.2). 39% of all participants (*n* = 7) were currently pregnant, most of whom were experiencing their first pregnancy (71%). Overall, 72% had been pregnant previously, and 22% reported a prior pregnancy loss. Among participants who responded to questions about GDM (*n* = 16), 11% reported a prior diagnosis, 61% reported no history, and 17% were unaware of their GDM status. 50% of all participants (*n* = 9) were enrolled in the Social Health Insurance Fund (SHIF) and had paid their premiums, indicating active coverage under Kenya’s national primary health insurance. Table 1 summarizes these characteristics.

**Table 1:** Demographic and health characteristics of study participants

Characteristic	*N* (%)	Mean (SD)
Age (years)	19–44	26.6 (7.2)
Marital Status		
Married	16 (88.9)	-
Single (Never Married)	1 (5.6)	-
Divorced or separated	1 (5.6)	-
Education Level		
Pre-Primary or None	1 (5.6)	-
Primary School	7 (38.9)	-
Secondary/ Post-Primary/ Vocational	7 (38.9)	-
College Level or Higher	3 (16.7)	-
Partner’s Education Level		
Primary School	6 (33.3)	-
Secondary/ Post-Primary/ Vocational	9 (50.0)	-
College Level or Higher	2 (11.1)	-
N/A	1 (5.6)	-
Occupation		
Unemployed	10 (55.6)	-
Contract/Temporary Worker	1 (5.6)	-
Self-Employed	7 (38.9)	-
Health Insurance Ownership		
Yes	9 (50.0)	-
No	9 (50.0)	-
Currently Pregnant		
Yes	7 (38.9)	-
No	11 (61.1)	-
First Pregnancy		
Yes	5 (27.8)	-
No	13 (72.2)	-
History of Gestational Diabetes Mellitus (GDM)		
Yes	2 (11.1))	-
No	11 (61.1)	-
N/A	3 (16.7)	-
Missing	2 (11.1)	-
History of Hypertension (HTN)		
Yes	2 (11.1)	-
No	11 (61.1)	-
N/A	3 (16.7)	-
Missing	2 (11.1)	-
Lost Child in Pregnancy		
Yes	4 (22.2)	-
No	9 (50.0)	-
N/A	5 (27.8)	-

Percentages are calculated using the total sample size (*N* = 18). “*N/A*” Not applicable indicates items not applicable (e.g., pregnancy loss in participants pregnant for the 1^st^ time) or participant uncertainty about prior diagnosis. “Missing” indicates participants who did not respond to the item

The number of facilitators and barriers to engagement in the STRiDE-GDM screening varied across all 18 participants. Knowledge, Environmental Context and Resources, Motivation, Intentions and Goals and Beliefs about Consequences were the most discussed domains, whereby Reinforcement, Beliefs about Capabilities, Behavioral Regulation and Skills domains were discussed least. Most participants reported more barriers than facilitators in relation to structural factors and gaps in knowledge. Facilitators were most often tied to personal motivation and the belief that GDM screening would lead to positive outcomes such as early diagnosis and management. This variability is reflected in Table 2, which summarizes the salience of TDF domains across all interviews.

**Table 2:** Identification of important TDF domains

TDF Domain	Themes	Sub-themes	Importance Criteria				Overall Importance
			Frequency		Conflicting Beliefs	Influence on Behavior	
			Barriers (*n*)	Facilitators (*n*)			
Knowledge	Lack of GDM knowledge	Risks; Lifestyle behaviors for prevention; postpartum persistence, GDM screening processes	42	19	Yes	High	High
	Low awareness on GDM screening	Importance of GDM screening					
	Misinformation and myths	Incorrect beliefs about risks and causes of GDM					
Environmental Context and Resources	Access to care	Cost; transport; travel times; availability of screening services	34	15	No	High	High
	Facility Care	Medication access; patient-provider relationship; fear; waiting times					
	Competing priorities	Caregiving, farming and employment					
	Access to GDM information	Peer group education; community education					
Motivation, Intention & Goals	Desire for good health	Personal and offspring; GDM health status	2	45	No	Moderate-High	High
	Stability of intentions	Peer influence					
	Access and convenience factors	Free screening; Scheduled screening; Action planning					
Beliefs about Consequences	Perceived benefits	Maternal; Fetal; Community health	15	25	Yes	High	High
	GDM Stigma						
	Severe anticipated outcomes	Death; lifestyle behavioral changes					
	GDM can be treated	GDM can be managed					
	Religious Beliefs	God determines GDM diagnoses					
Social Influences	Social support	Spousal; Family; Peers	12	18	Yes	Moderate-High	Moderate-High
	Health professionals	Trust in CHPs; Clinicians recommendations					
Emotions	Negative Emotions	Fear of screening and a positive diagnosis; Stress; Worry; fear of losing peer-support	17	13	Yes	Moderate-High	Moderate-High
	Positive Emotions	Relief; peer group support					
Social Professional Role & Identity	Maternal role	Prioritizing personal health to fulfill maternal & family responsibilities	10	18	No	Moderate-High	Moderate-High
	Community roles	Being a role model; health promoter					
	Professional roles	CHPs and clinicians promoting GDM screening; influencing uptake					
Reinforcement	Prior experiences	Negative care experiences	10	3	No	Low	Low
	Education sessions	Provided in peer groups; repeated often					
Beliefs about Capabilities	Perceived ability to attend GDM screening and referrals	Ability to handle competing priorities; coping with GDM	2	3	Yes	Low	Low
	Perceived ability to manage GDM	Confidence in treatment adherence; ability to adopt new lifestyle behaviors					
Behavioral Regulation	Seeking GDM information	CHP - provided	0	4	No	Low	Low
Skills	Clinician screening competence	OGTT screening; Communication skills	1	1	No	Low	Low
Total			**145**	**164**	**309**		

Frequency: relative number of coded responses; Conflict: number coded as conflicting beliefs; Influence on behavior: how likely coded responses could impact engagement in screening. Overall importance: combined assessment of the three importance criteria

Beliefs were identified across multiple TDF domains and classified as important based on salience criteria described in the Methods section. Behavioral regulation, Skills, Reinforcement, and Beliefs about Capabilities were classified as low-importance domains due to few distinct belief statements and substantial overlap with other domains captured through double coding. These domains are therefore not discussed in detail below.

#### Knowledge domain

Three identified themes mapped to the knowledge domain, lack of knowledge, lack of awareness and misconceptions about GDM. Within this domain, participants commonly discussed barriers such as limited understanding of risks or preventive factors compared to facilitators such as importance of GDM screening.

##### GDM knowledge/awareness 

Prior health education on chronic diseases delivered by CHPs in the peer groups improved knowledge and awareness, which motivated participation in screening,

*“I have heard it is good*,* especially for pregnant mothers…. to be tested early to help manage or to prevent yourself from the risk of getting diabetes”* [P14].

Other participants expressed that the knowledge gained motivated them to screen and adopt healthier behaviors for risk reduction. This common view was expressed as follows,

*“We were educated that there are foods which you can’t eat*,* like those enriched with sugars” [ P8] and*,* “on doing exercise”* [P4].

##### Misconceptions about GDM 

Though some GDM education had been provided in the peer groups, majority of the responses suggested that *Chamas* participants still had limited understanding or misconceptions about GDM. This limited understanding hindered participation as explained by one participant,

*“I was told to test I refused and said I don’t drink sugary tea”* [P15].

##### GDM Knowledge 

Participants understanding of the frequency of GDM screening was also low. Many believed that it should be done monthly, with one participant sharing that, “*I used to come and test in Chamas group’s month to month”* [P4]. Further questions arose regarding postpartum persistence of GDM if they had a positive diagnosis,

“*Like right now if I have diabetes and I get to deliver. Will it be affecting me onwards?”* [P5].

Building on these findings, feasible strategies to address these barriers in the planned pilot, include providing GDM peer group education sessions and informational material to raise screening awareness, clarify screening procedures and correct minor misconceptions.

#### Motivation, intentions and goals domain

Within the Motivation, Intentions and Goals domain, three main themes were identified, desire for good health, stability of intentions and factors that improve access and convenience for GDM screening.

##### Desire for good health 

Participants had a strong desire to know about their health status to the extent that some were willing to pay out-of-pocket costs to cover GDM screening and follow-up care. This common view was expressed as follows:

*“I would accept to pay because I would want to know about my diabetes”* [P4].

This was further emphasized by another participant,

*“When I have money or insurance I will pay for the test because of my health. If I will not sacrifice for it*,* I am the one who will be affected”* [P1].

For majority of the participants, knowing their health status was critical to inform improved health for themselves and their children.

##### Stability of intentions 

Participants were highly motivated to get screened, such that they prioritized their health status over family or peers’ opinions. One participant shared,

*“No*,* I could not listen to [my peers’] opinion because I am the one carrying my baby*,* so I just care for myself”* [P10].

Factors that improve access and convenience of GDM screening, were highlighted as important in supporting action planning and intention to attend GDM screening. Participants suggested that implementers should schedule and communicate GDM screening dates in advance so that they could plan appropriately. This theme was summarized by a participant who said,

*“So*,* if you want to test us you also inform us on the date*,*”* [P16].

Despite facing financial barriers to accessing health care services, participants demonstrated strong motivation and suggested that providing free, community-based GDM screening would motivate them to get screened. One participant shared,

“*It benefits us when you have offered free test without payment to us in the group of Chamas*” [P2].

To support this level of motivation in the upcoming pilot, strategies could include emphasizing the benefits of screening for both mother and child during the group education sessions. Improving access and convenience of the STRiDE-GDM screening could also reduce structural barriers and make participants more likely to act on their intentions.

#### Environmental, context & resources domain

Within this domain, participants described a range of external factors that influenced uptake of GDM screening. Overall, more barriers than facilitators were identified and captured under the following themes: access to care, facility care, competing priorities and access to GDM information.

##### Access to care 

Majority of respondents discussed access to GDM screening challenges, with cost, transport expenses, travel time, and availability of screening services emerging as sub-themes. As such, participants commonly shared that free or nearby screening services especially when brought to them in peer groups increased uptake to screening.

*“They brought the test to our group. We didn’t have to go far*,* and that made it easier”* [P2].

In contrast, high transport costs and long distance to facilities discouraged participants from screening as shared by two participants,

*“The challenge is transport to go to the clinic and whereby the facility is far”* [P2].

*“I did not manage to go because I did not have money for transport to go”* [P15].

Though many participants recognized the importance of GDM screening and were willing to get screened, they chose not to pursue it due to financial barriers, with one saying,

*“It will be difficult because of not having the capability of paying for the test but logically I accept to be tested”* [P2].

On GDM referral completion, financial constraints further limited participant’s ability to pay for GDM follow-up care if they got a positive diagnosis as one participant explained,

*“And then I was thinking if I get there*,* I would be asked for money for treatment and I didn’t have any”* [P15].

##### Facility & community-based screening services 

Majority of the participants who preferred facility-based GDM screening believed that a positive GDM diagnosis required medication which they perceived would be easier to access in a hospital setting as one participant explained,

*“[Facility is better than community] because in the hospital it is faster you are given medicine”* [P16].

In contrast other women were ambivalent about screening in facilities due to multiple reasons such as fear, transport, distance and poor patient-provider relationship as explained by one participant,

*“Many of us when pregnant fear to go to the hospital to be tested” [P18]*,* “doctors shout at people in the facility”* [P18].

Long waiting times, especially in a fasted state within medical facilities, further decreased participants’ motivation to screen after traveling long distances. As two participants explained,

***“****You have come all the way from home… you stay up to 2 pm you have not taken anything*” (P17] and,

“*Also*,* when we reach the facility*,* there is a long queue so that lowers our morale to go”* (P14).

##### Competing priorities 

Caregiving, farming, and employment were often prioritized over health seeking behaviors like GDM screening especially in households experiencing food insecurity. This was strongly expressed by one participant,

*“You might not have money [for GDM care] and maybe the money you have is to feed the children you have. So*,* you see when I go to use the money in treatment the children might sleep hungry. So*,* the challenge is just lack of money and hunger*” [P15].

A similar perspective emerged in the comment,

*“When someone is digging or supposed to be in the market for that day*,* it [GDM screening] feels like time wasting”* [P10].

##### Accessibility of GDM information/materials 

Women expressed interest in having accessible GDM information to reinforce their understanding of symptoms and prevention strategies. As one participant expressed,

*“Come and give us more information about GDM”* [P9].

In the upcoming pilot, feasible strategies to reduce structural barriers such as travel and facility costs include delivering the STRiDE-GDM screening via *Chamas* maternal community- based peer groups as originally planned. CHPs will also collaborate with participants to create flexible GDM screening schedules that accommodate work and childcare responsibilities.

#### Beliefs about consequences domain

Within this domain, participants, reflected on outcomes of GDM screening, with majority highlighting positive benefits such as improved maternal and fetal health, while others were concerned about negative consequences including stigma and fear of a positive diagnosis. Four themes mapped to this domain, perceived benefits, stigma, religious beliefs, severe anticipated outcomes, and hope around the fact that GDM can be treated.

##### Maternal, fetal, and community health benefits. 

Majority of the participants believed that GDM screening and early diagnosis would reduce complications and mortality for both mother and child, thus a motivator to participate in screening. A common view was expressed as follows:

*“In case you are found with diabetes you are directed to start medication early*,* so that it might not affect the baby and also the mother’s health is not deteriorated”* [P1].

Participants also believed that being healthy and having healthy children contributed to improved community health as stated by another participant,

“*The benefit [of GDM screening] is that when we are healthy*,* the place we stay will also be of good health”* [P8].

##### GDM can be treated 

Participants felt hopeful that that a positive diagnosis through screening would guarantee access to GDM treatment for them to get well. As one participant explained,

*“First we will be tested to see which level of sugars and then be enrolled for treatment” [P8].*

##### Severe anticipated outcomes

In contrast, other women were more concerned with severe outcomes they associated with GDM diagnosis such as death of mother and baby which discouraged them to seek screening. As one participant shared,

*“It [GDM] is dangerous*,* it kills and maybe when you have it and you breastfeed the baby it can go through breast milk to the baby….I understand it is a disease that can cause a pregnant mother to lose pregnancy” [P1].*

This participant’s belief was grounded in a misconception that GDM is transmitted through breastmilk.

There was also a hesitation to adopt new lifestyle behaviors following a GDM diagnosis which hindered participation in screening. One participant shared,

“*I normally eat sugars…. you know when you are used to sugars*,* if you are not used to using it is just okay. But when they tell you not to use it…. it’s a problem”* [P12].

##### Stigma 

GDM being a chronic disease is associated with stigma in this setting. As such, stigma concerns discouraged participation in screening as women feared alienation in the community if they got a positive diagnosis. As one participant explained,

*“One thing is that others get scared or fear discrimination and stigmatization in the community”* [P16].

As such, some participants shared concerns about privacy when screened in peer groups. These participants felt uncomfortable screening in their peer groups expressing fear that a positive GDM diagnosis might be disclosed to other community members without their permission resulting in stigma or alienation. This was reflected in the comment,

*“You know when we are in groups when being tested everyone will know that I have diabetic disease”* [P16].

Not everyone agreed, with some participants expressing a preference for routine GDM screening to be provided in their peer groups. These participants felt that group screening had the potential to reach and benefit more women. As one participant explained,

*“[GDM screening should be routinely provided in groups] as it will help many women to know their health in diabetes”* [P4].

Religious Beliefs shaped some participants’ decision to screen for GDM. Some participants described that negative diagnoses were determined by God which undermined the perceived value of GDM screening. This was reflected in the comment,

*“Someone prays that he or she is not positive with diabetes disease…I say God help me that it may not affect me”* [P16].

These contrasting beliefs highlight both the perceived risks and potential benefits of the proposed STRiDE-GDM screening. In the upcoming pilot, feasible strategies to address participants concerns about GDM screening include educating participants on what a GDM diagnosis means including outcomes and ensuring timely referral to the local health care facility for care. Some barriers, such as stigma or religious beliefs are acknowledged but cannot directly be addressed in the pilot.

#### Social influences domain

Within this domain, participants identified interpersonal relationships with spouses, family members, peers, and health care providers as key influences on GDM screening uptake. Overall, participants more frequently highlighted supportive relationships that encouraged them to screen, compared to lack of supportive relationships.

##### Spousal and family support 

Participants consistently described their husbands as gatekeepers to engagement in health care behaviors such as GDM screening. A common view was expressed as follows:

*“I will not disagree to test; it will depend on my husband. but when you go there the husband may say get out of that group and stay at home”* [P15].

##### Peer-support and group encouragement 

Positive and negative opinions of women enrolled within the *Chamas* peer groups influenced individual’s participation in GDM screening. Regardless, majority of the participants were adamant that they would not be swayed. A common view was expressed as follows,

*“No*,* it won’t hinder me from doing a diabetes test because I am the one who will suffer if I follow negative opinions”* [P17].

Participants also appreciated encouragement from their peers which increased their confidence to pursue GDM screening. This theme was summarized by a participant who said,

*Testing should be done in groups…. because we will be able to help each other*,* It makes you have hope and that you are not alone with it and you can be able to advise each other”* [P17].

##### Support from health care providers and CHPs 

Involvement of health care providers such as doctor’s who are highly esteemed and CHPs who are trusted in the community motivated participants to engage in GDM screening. Several participants noted,

“*If our [CHPs] say we*,* do it*,* we just hear*,* obey and come*” [P7].

A similar perspective emerged in the comment,

*“We were being tested by a doctor” … and “If the doctor requests me to get GDM screening*,* I will accept to go and do the test”* (P16),

Building on these findings, feasible strategies to enhance supportive influences in the STRiDE-GDM pilot include leveraging CHPs to facilitate participant recruitment and screening and peer groups to facilitate participation as originally planned. Family-related barriers are acknowledged but cannot be directly addressed at this stage.

#### Emotions domain

Within this domain, participants identified negative emotions such as fear, stress and worry more frequently than positive emotions such as hope and relief and how they shaped decisions about GDM screening. Two themes emerged in this domain, negative and positive emotions.

##### Negative emotions 

Fear was the most common emotion linked to the screening procedure itself, positive diagnosis, or perceived discrimination, which often discouraged women from testing and completing referrals. As one participant explained,

*“You must be worried…. you start asking yourself if I have diabetes what will I do?”* [P17]. As another stated, *“You get stressed*,* depressed and so many questions about how and why. Like when you go for delivery*,* what will happen if the sugar levels go high?”* [P14].

Peer-support was frequently described as a strong facilitator of GDM screening. However, due to a misconception that GDM screening is performed more than once, some participants feared attending their peer group meetings consistently. As one participant shared,

*“You can even avoid going for Chamas because when you come the doctor might test you again on the glucose”* [P16].

A similar perspective, emerged in the comment,

*“They may disclose [the positive GDM status] and that one may bring shame”* [P10].

This participant was concerned that a positive GDM diagnosis could lead to shame which may result in isolation causing participants to avoid coming to *Chamas* groups.

Stress about how participants would manage a positive diagnosis, and potential effects on the fetus reduced motivation to screen. This was reflected in the comment,

“*Disadvantage may be when you are tested and found positive with diabetes you may start having stress resulting in the baby being affected. Like you may lose the baby through miscarriage”* [P16].

In this case, the participant’s stress was not about the screening itself, but the anticipated perceived severe outcome of testing positive.

In contrast, negative diagnoses brought positive feelings such as relief to the participants. As one participant explained,

*“You get excited when you get to know your results that you don’t have diabetes”* [P4].

In the upcoming pilot, feasible strategies to address emotional barriers include providing clear explanations of the STRiDE-GDM screening process and implications of a positive GDM diagnosis during educational group sessions to reduce fear and anxiety.

#### Social professional role and identity domain

Within this domain, participants reflected on how social roles and expectations related to motherhood along with health care provider involvement influenced participants’ decisions to engage in GDM screening. Three themes emerged in this domain, maternal role, community role, and health professional role.

##### Motherhood responsibilities to prioritize care of the child and the family 

Participants felt motivated to be healthy so that they could protect their babies. This was reflected in the comment,

“*As a mother*,* it is my duty to make sure I don’t put my child at risk. That’s why I accepted the test”* [P12].

Perceived motherhood responsibilities did not stop with the baby, however, was evident in the broader family context as well. As another participant stated,

“*It is important to test because the family depends on me”* [P12].

##### Community role 

Participants described wanting to be role models and share their health seeking experiences. As such they assumed positive roles as health promoters. As one participant explained,

***“****Once you are tested*,* you are able to have strength to inform others to visit a hospital for them to know their health status in diabetes”* [P8].

##### Role of medical providers in GDM management 

CHPs and clinical officers were seen as influential in guiding GDM screening decisions by providing community education and managing GDM. As one participant explained,

*“CHPs taught us about diabetes testing in our groups” [P15]*.

As another stated,

*“Diabetes doctors will bring [GDM] down slowly by slowly it will come down*” [P18].

In the upcoming pilot, feasible strategies to leverage these influences include highlighting the maternal benefits of screening during group education sessions to reinforce motivation to engage in the STRiDE-GDM screening.

## Discussion

### Summary of main findings

Guided by the TDF, this study explored psychological, social and contextual facilitators and barriers to STRiDE-GDM screening among women in *Chamas* peer groups in rural western Kenya. Our findings demonstrate that women’s decisions to engage in GDM screening using the STRiDE tool was shaped by a combination of individual beliefs, social influences, and health system level factors. Interpretation of these findings is grounded in the specific study context of *Chamas* peer groups in rural western Kenya, where majority of participants were married with varying levels of education, employment and insurance coverage. Given the limited availability of GDM screening implementation studies in Kenya, findings are discussed primarily within this setting, with evidence from other chronic disease screening studies in SSA used to triangulate and contextualize results rather than to generalize broadly.

At the individual level, participants reported gaps in GDM knowledge and low perceived risk, which reduced motivation to engage in screening. Fear of a GDM diagnosis and potential negative consequences further hindered screening engagement. These concerns were particularly salient in this rural western Kenya context, where women often interact with formal health services primarily during pregnancy and may have limited opportunities for structured health education outside ANC. Similar knowledge related barriers have been reported in cervical cancer, diabetes and hypertension screening studies broadly in SSA [31–36], in which low health literacy especially within rural settings and fear of diagnosis reduces uptake of screening. Cultural, societal and religious beliefs were also found to shape perceptions of chronic disease, contributing to stigma and fear surrounding utilization of Maternal and Child Health care services (MCHS) in Kenyan studies [18, 35, 37]. Though we reference studies from other chronic diseases broadly within SSA, they are mainly used to contextualize behavioral determinants but not generalize the experiences observed among *Chamas* participants. Within this study population, peer group education sessions led by CHPs emerged as important enablers to GDM screening engagement. Participants felt that these peer group sessions provided a trusted space where they could ask questions, clarify misconceptions, normalize testing, and reduce fear through shared experiences. However, privacy and confidentiality concerns also arose. Unlike prior SSA studies, where such concerns center on patient-provider interactions [38, 39], participants in this study reported these concerns within the peer group context itself. While many participants valued the educational and emotional support offered in *Chamas*, some feared that a positive result could be disclosed within the group, resulting in stigma which hindered screening engagement. These findings highlight the potential of community-based peer groups to fill knowledge gaps that formal health systems may not consistently address. However, concerns about privacy and confidentiality suggest that even when improving knowledge, it must be done carefully to ensure women feel safe and supported when engaging in GDM screening.

Building on these knowledge-related determinants, participants engagement with the STRiDE-GDM screening was also shaped by social influences including spouses, health care providers and peers. Participants described how marital dynamics, peer concerns, and community perceptions influenced their willingness to screen for GDM with fear of disclosure and stigma acting as barriers. These dynamics were relevant in our study given that most participants were married and lived in close-knit rural communities. In rural communities like the one studied, privacy maybe limited and spousal support is important when making health care decisions. This is consistent with prior cervical cancer screening studies within SSA which demonstrated that spousal support is strongly linked to health care seeking behavior [38]. Studies in Kenya, also reported that husbands directly influenced whether or not their wives accessed MHCS primarily due to women’s financial dependence on their spouses [35, 40]. This dynamic is especially salient in our sample where at least half of the participants were unemployed and lacked active health insurance coverage. These conditions highlight why spousal support and approval were particularly important in this population. Health care providers and CHPs also strongly influenced engagement in GDM screening, as their guidance was trusted and highly valued. Consistent with studies from Kenya, CHPs act as a bridges between communities and formal health services to promote testing, provide health education, and facilitate care uptake [41–43]. This underscores the critical role of trusted CHPs in facilitating screening behaviors in rural Kenyan communities. In contrast, although peer groups offered education and a supportive space, participants reported that peer influence did not strongly affect their screening decisions.

In summary, our findings highlight multiple determinants of women’s engagement in STRiDE-GDM screening, including knowledge gaps, mixed emotions, stigma, social influences, and structural barriers. These findings demonstrate that both individual, interpersonal and environmental factors should be considered when designing contextually appropriate screening interventions. Based on these insights, the proposed STRiDE-GDM implementation strategy pilot will be modified to address feasible barriers before piloting, with the goal of improving successful implementation and uptake. Persistent challenges such as stigma, emotional concerns, women’s economic dependence, and gaps in policy and health system financing will not be addressed prior to the pilot as they require longer-term investment. Addressing these persistent challenges will require longer-term efforts, such as engaging government stakeholders to discuss supportive policies focused on accessible, available and affordable GDM services. It is also necessary to engage spouses and community members through GDM awareness and dialogue which can help shift mindsets and provide social and emotional support that motivates women to participate in screening. Building on these findings, the following section, outlines short-term, strategies designed to address modifiable barriers identified in this study which will inform the subsequent STRiDE-GDM pilot.

### Planned implementation responses

Across the salient TDF domains, identified in this study, planned implementation strategies focused on addressing modifiable barriers to support women’s engagement in the STRiDE-GDM screening pilot study. While all the domains provided insight about factors that would influence behavior, strategies were prioritized for the Knowledge and Environmental Context & Resources domains, which were considered most feasible to target prior to the pilot.

Within the Environmental Context & Resources domain, structural barriers including transport costs and long facility wait times were identified as hindering engagement to GDM screening. To address these barriers, the planned implementation strategy involves delivering the STRiDE-GDM screening in *Chamas* peer groups to reduce travel and facility-related costs. We also plan to provide flexible scheduling to accommodate work and childcare responsibilities. By attending to the *Chamas* participants in their home settings, we expect to improve access to GDM screening.

Within the Knowledge domain, gaps and misconceptions regarding GDM risk and importance of screening limited engagement. To target these barriers, we plan to provide peer group community educational sessions on GDM, causes, common signs & symptoms, prevention and management. We also plan to develop contextualized take-home pamphlets translated into Swahili which have accessible GDM information including phone numbers for trained CHPs, and the study team for any questions or concerns. CHPs will be provided with ongoing training to raise community awareness about GDM and other crosscutting conditions that affect maternal and fetal health. Increasing awareness, is likely to encourage greater participation in the STRiDE-GDM screening pilot. As part of raising awareness, we will also develop and distribute easy to understand flyers that will be housed in each peer group coupled with education sessions during routine group sessions to normalize screening.

These strategies informed by the qualitative findings will inform the subsequent feasibility pilot of the STRiDE-GDM screening approach in *Chamas* groups.

### Strengths of the study

Key strengths of this study were using the TDF and interviewing multiple stakeholders. Our study employed the TDF; a theory-driven, systematic approach which reinforced study validity. Multiple stakeholders (CHPs and clinical officers) in addition to women in *Chamas* peer groups were interviewed to get a deeper understanding of implementation determinants from a diverse perspective. Inclusion of CHPs was crucial to inform implementation of our STRiDE-GDM screening strategy which relies on community engagement to capture participants in early pregnancy. Additionally, the health care practitioners provided valuable insight into the current infrastructure for GDM service delivery in local health care facilities. Findings from other stakeholders (CHPs and Clinicians) will be reported in a later paper.

### Study limitations

This study may have lacked geographic, ethnic, and socio-economic diversity as participants were all members of similar community-based peer groups (*Chamas*) in rural western Kenya. As such generalizability of findings may be limited. Participants’ exposure to risk-stratified GDM screening varied. Some postpartum women reflected on prior routine care experiences while other participants responded to a described workflow not yet implemented. This may have influenced responses but also enabled exploration of both experiential and anticipated implementation barriers. Social desirability bias may have also influenced participant’s responses, especially as it relates to individual intent to screen for GDM. Though consenting to the study was voluntary, participants were offered meal and transport reimbursements which may have contributed to bias. We tried to mitigate this bias, by recruiting participants from multiple *Chamas* peer groups representing different stages of the perinatal continuum; those pregnant for the first time, those who had been pregnant before and those who had just given birth.

### Implications of these findings

Findings from this study highlight that participants from *Chamas* peer groups value support from CHPs and guidance from facility-based health care providers in adopting health- seeking behaviors such as GDM screening and management. This implies that integrating community and facility health care service delivery models can enhance access to care. By initiating the screening process within the community, we can improve access to care and promote health equity. Improved access to care is facilitated by eliminating the need to travel to receive GDM screening. We also believe that expanding access to GDM screening is expected to improve health outcomes via early diagnosis and referrals.

At a health policy level, implementing long-term routine GDM screening requires government buy-in specifically through adequate funding for the national health insurance program that benefits economically disadvantaged rural communities. Including a community-facility based GDM screening strategy in the ante-natal care package covered by the national health insurance program could address financial barriers, enhance accessibility, and encourage health seeking behaviors.

## Conclusion

This qualitative study explored barriers and facilitators of implementing the STRiDE-GDM screening strategy within community-based peer groups (*Chamas*). Our findings suggest that *Chamas* peer groups may offer an acceptable and accessible setting for implementation of the STRiDE-GDM screening in rural western Kenya where women experience difficulties in accessing affordable and quality health care consequently leading to poor GDM-related outcomes. This study found that most *Chamas* participants were highly motivated to screen for GDM to understand their health status and improve health outcomes for themselves and their offspring. Despite this motivation, they emphasized the need for greater personal, social and financial support. The STRiDE-GDM screening strategy must therefore address the identified modifiable barriers for successful implementation and meaningful impact to be realized.

## Supplementary Information

Supplementary Material 1.

## Data Availability

Data from this study contains information that may compromise the confidentiality and privacy of participants. However, requests made to the corresponding author are considered appropriate. Requests can be submitted to the corresponding author at rogumbo@purdue.edu.
