# Supplementary material for: Implementation determinants of risk-stratified gestational diabetes mellitus screening in community-based women’s peer groups in rural western Kenya
Source: BMC Pregnancy Childbirth. 2026 May 18;26:754. doi: 10.1186/s12884-026-09081-6 (PMC13352656; doi:10.1186/s12884-026-09081-6)
Supplement: Supplementary file 1 — Supplementary Material 1. [file 12884_2026_9081_MOESM1_ESM.docx]

Supplementary Material

**Supplementary file 1. Key stakeholders and their roles in the STRiDE-GDM implementation**

| **Stakeholder** | **Definition** | **Role in implementation of STRiDE-GDM screening** |
| --- | --- | --- |
| ***Chamas* participants** | Pregnant and postpartum women ≥ 18 years enrolled in *Chamas*. | Consent and attend the STRiDE-GDM screening, complete facility referrals if diagnosed with GDM. |
| **Community Health Promoters** | Adults ≥ 18 years with a high school diploma, trained to deliver health promotion and facilitate access to health care facilities. | Identify *Chamas* participants <20 weeks’ gestation, use the STRiDE tool to stratify GDM risk and support referral for GDM patients |
| **Clinical Officers** | Mid-level health care providers | Diagnostic OGTTs, treatment for women diagnosed with GDM |

The key stakeholder’s highlighted in *Table 1*, were recruited to capture diverse perspectives on determinants of the proposed STRiDE-GDM screening approach.

**Supplementary file 2. In-Depth Interview guide.**

**Study Title**: Barriers and Facilitators to Implementing the STRiDE tool to screen for Gestational Diabetes Mellitus (GDM) in community-based peer groups (*Chamas*)

Semi-structured Interview Questions guided by the Theoretical Domains Framework to understand coginitive, behavioral and environmental determinants that could influence implemementation of the STRiDE-GDM screening strategy in community-based peer groups (*Chamas*)

Interview date: ___/___/___ (Date/Month/Year)

Start time: __________________

Introduction: Interviewer to introduce him/herself. Explain study procedures and details to participants, including audio recording, confidentiality, and rights to refuse participation. Obtain written informed consent.

| **QUESTION NO.** | **QUESTION** | **PROMPTS** | **COMMENTS** |
| --- | --- | --- | --- |
| **KNOWLEDGE** | | | |
| 1 | Please describe what you understand by Gestational Diabetes Mellitus (GDM) | *What were you told in the past about GDM?*  *What puts a woman at risk of GDM?*  *What happens when a woman has GDM?* |  |
| 2 | Can you tell me what you know about GDM testing which occurs during pregnancy? |  |  |
| 3 | Have you ever been tested for GDM or heard from others what happens during GDM testing? | *If yes, when? And where?*  *What difficulties did you face in being screened?*  *What was helpful about being screened?* |  |
| 4 | What do you understand about the p -GDM screening proposed in *Chamas*’*s* | *What do you understand needs to be done to get the GDM screening offered in Chamas?* |  |
| **ENVIRONMENT, CONTEXT AND RESOURCES** | | | |
| 5 | Can you describe how easy or difficult it is for people to get tested for GDM in your area | *Where do people get tested?*  *Do you have to travel far to get tested?* |  |
| 6 | How would you pay for GDM testing if the doctor requires you to get it? | *Insurance?*  *Out-of-pocket payments?*  *Can not afford to pay?* |  |
| **SOCIAL INFLUENCES** | | | |
| 7 | Do the opinions of people important to you on the STRiDE-GDM screening process affect your participation? | *Spouses, family-members, CHPs, Clinical officers, Peers in Chamas* | **note: this subprompt could also be coded as TDF domain Emotions, Social Professional Role & Identity* |
| 8 | Are there any conflicting beliefs amongst your peers about the STRiDE-GDM screening process? | *Peers in Chamas* |  |
| **BELIEFS ABOUT CONSEQUENCES** | | | |
| 9 | What do you think are the perceived disadvantages of screening for GDM in *Chamas*? | *To what extent do you feel these views are shared by your peers in Chamas?* | **note: this subprompt could also be coded as TDF domain Social Influences* |
| 10 | What do you think are the benefits of screening for GDM in *Chamas*? | *To what extent do you feel these views are shared by your peers in Chamas?* | **note: this subprompt could also be coded as TDF domain Social Influences* |
| **MOTIVATION AND GOALS** | | | |
| 11 | Can you tell me what might make you want to be screened for GDM in *Chamas*? |  | **note: this subprompt could also be coded as TDF domain Social Influences/ Beliefs about Consequences* |
| **REINFORCEMENT** | | | |
| 12 | Can you describe any experiences you’ve had with any kind of health screening, like GDM or other clinic testing that have influenced whether or not you could get tested again in the future |  | **note: this subprompt could also be coded as TDF domain Environmental Context & Resources* |
| **INTENTIONS** | | | |
| 13 | On a scale where zero means “none of the time” and five means “always”, how likely are you to get screened for GDM in *Chamas*? | *Can you explain/elaborate your answer.*  *What of the next pregnancy?* |  |
| **OPTIMISM** | | | |
| 14 | How optimistic or pessimistic are you that GDM screening in *Chamas* will improve care for pregnant women like you? |  |  |

**Supplementary file 3 : COnsolidated criteria for REporting Qualitative research (COREQ) checklist**

| **Topic** | **Item No.** | **Guide questions/description** | **Manuscript**  **page no.** |
| --- | --- | --- | --- |
| **Domain 1. Research team and reflexivity** | | | |
| *Personal characteristics* | | | |
| Interviewer/facilitator | 1 | Which author/s conducted the interview or focus group? Interviews were conducted by co-authors HN, SC and EK who are trained research assistants with long-standing experience working with *Chamas* groups for many years. | P6 |
| Credentials | 2 | What were the researcher’s credentials? E.g., PhD, MD. Co-authors held academic degrees and were trained as research assistants; specific degree qualifications are not specified. | N/A |
| Occupation | 3 | What was their occupation at the time of the study? *Chamas* administrative Staff | P6 |
| Gender | 4 | Was the researcher male or female? Interview team had One male (HK) and two females (EK, SC). Analytic team had 3 females. | P6 |
| Experience and training | 5 | What experience or training did the researcher have? Extensive experience working with *Chamas* community groups and training in qualitative interviewing and research ethics. | P6 |
| *Relationship with participants* | | | |
| Relationship established | 6 | Was a relationship established prior to study commencement? Some interviewers had previously engaged some participants through community activities; however, none had a clinical care relationship | P6 |
| Participant knowledge of the interviewer | 7 | What did the participants know about the researcher? e.g., personal goals, reasons for doing the research. Participants’ were informed about the study purpose and the STRiDE-GDM screening process. They were also told that the interviews would focus on their experiences, beliefs and anticipated perceptions. | P6 |
| Interviewer characteristics | 8 | What characteristics were reported about the interviewer/facilitator? e.g., Bias, assumptions, reasons, and interests in the research topic . Interviewers were *Chamas* peer groups administrators and trained research assistants familiar with the local context and the *Chamas*, community-based peer groups. Interviewers were encouraged to document reflexive notes following interviews, and team debriefings occurred at the end of the interview day to allow reflection space on positionality and potential influence on data collection. | P7,8 |
| **Domain 2: Study design** | | | |
| *Theoretical framework* | | | |
| Methodological orientation and Theory | 9 | What methodological orientation was stated to underpin the study? E.g., grounded theory, discourse analysis, ethnography, phenomenology, content analysis. The study was underpinned by a hybrid inductive-deductive thematic analysis approach, guided by the TDF to identify facilitators and barriers. | P7 |
| *Participant selection* | | | |
| Sampling | 10 | How were participants selected? E.g., purposive, convenience, consecutive, snowball. Eligible *Chamas* participants were identified through purposive sampling with the help of an administrator who organizes the *Chamas* peer groups and Community Health Promoters. | P5 |
| Method of approach | 11 | How were participants approached? e.g., face-to-face, telephone, mail, email . *Chamas* participants who expressed interest and met inclusion criteria were subsequently recruited by CHPs in person or via phone calls. | P5 |
| Sample size | 12 | How many participants were in the study? 18 participants were in the study | P5 |
| Non-participation | 13 | How many people refused to participate or dropped out? Reasons? None of the participants that were approached refused to participate or dropped our during the study. | N/A |
| *Setting* | | | |
| Setting of data collection | 14 | Where was the data collected? e.g., home, clinic, workplace . Interviews were scheduled at the convenience of *Chamas* participants and conducted in their home setting, at nearby health dispensaries or hospitals close to their home. | P6 |
| Presence of non-participants | 15 | Was anyone else present besides the participants and researchers? No one was present besides the participants and researchers | N/A |
| Description of sample | 16 | What are the important characteristics of the sample? e.g., demographic data, date . 18 *Chamas* women were interviewed.  *Chamas* participants’ mean age was 26.6 years (SD=7.2). 39% of all participants (n=7) were currently pregnant, most of whom were experiencing their first pregnancy (71%). Overall, 72% had been pregnant previously, and 22% reported a prior pregnancy loss. Among participants who responded to questions about GDM (n=16), 11% reported a prior diagnosis, 61% reported no history, and 17% were unaware of their GDM status. 50% of all participants (n=9) were enrolled in the Social Health Insurance Fund (SHIF) and had paid their premiums, indicating active coverage under Kenya's national primary health insurance | P8, T1 |
| *Data collection* | | | |
| Interview guide | 17 | Were questions, prompts, guides provided by the authors? Was it pilot tested? Interviews were guided by a 13-question SSI topic guide informed by the TDF. To confirm that questions were understandable and relevant, the SSI guide was reviewed by the research team and a qualitative expert, then piloted for face validity with 3 *Chamas* participants. Revisions were made to simplify complex questions, improve clarity and shorten the length of the interview | P6, S2 |
| Repeat interviews | 18 | Were repeat interviews carried out? If yes, how many? No repeat interviews were carried out. | N/A |
| Audio/visual recording | 19 | Did the research use audio or visual recording to collect the data? The research used audio recordings to collect the data. | P6 |
| Field notes | 20 | Were field notes made during and/or after the interview or focus group? interviewers were encouraged to take notes during and after the interview, reflecting on and documenting their perceptions or reactions. | P7 |
| Duration | 21 | What was the duration of the interviews or focus group? Mean interview duration was around 20 to 25 minutes | P6 |
| Data saturation | 22 | Was data saturation discussed? Yes. Data were collected from all 18 participants, and the emergence of new insights was monitored throughout the analysis. No new beliefs or themes emerged in the final interviews, confirming that thematic saturation was achieved | P6 |
| Transcripts returned | 23 | Were transcripts returned to participants for comment and/or correction. No, transcripts were not returned to participants for review or correction. | N/A |
| **Domain 3: analysis and findings** | | | |
| *Data analysis* | | | |
| Number of data coders | 24 | How many data coders coded the data? Data were coded by the analytic team, consisting of two trained research assistants and post-doctoral fellow with expertise in qualitative methods. | P7 |
| Description of coding tree | 25 | Did authors provide a description of the coding tree? A codebook was developed and used by the analytic team to guide coding. It is not provided as supplementary material. | P7 |
| Derivation of themes | 26 | Were themes identified in advance or derived from the data? Themes were identified using an inductive-deductive approach | P7 |
| Software | 27 | What software, if applicable, was used to manage the data? Microsoft Excel was used to manage the data. | P7 |
| Participant checking | 28 | Did participants provide feedback on the findings? Participants did not provide feedback on the findings. | N/A |
| *Reporting* | | | |
| Quotations presented | 29 | Were participant quotations presented to illustrate the themes/findings? Was each quotation identified? e.g., participant number. Yes; quotations are presented with participant identifiers. | P11-17 |
| Data and findings consistent | 30 | Was there consistency between the data presented and the findings? Yes; findings are consistent with data presented. | P17-19 |
| Clarity of major themes | 31 | Were major themes clearly presented in the findings? Yes; salient domains identified from the data are clearly presented in the findings. | P10-11, T2, |
| Clarity of minor themes | 32 | Is there a description of diverse cases or discussion of minor themes? Minor themes or less relevant domains are acknowledged but not discussed. | P9, P11 |

P: page number(s), S: supplementary materials section; T: in-text table number
